# Detection of vaginal lactobacilli as probiotic candidates

**DOI:** 10.1038/s41598-019-40304-3

**Published:** 2019-03-04

**Authors:** Alessandra Pino, Emanuela Bartolo, Cinzia Caggia, Antonio Cianci, Cinzia L. Randazzo

**Affiliations:** 1 Department of Agricultural, Food and Environment, University of Catania, Catania, Italy; 2 Department of General Surgery and Medical Surgical Specialties, Gynecological Clinic, University of Catania, Policlinico Universitario, Catania, Italy

## Abstract

The vaginal microbiota of healthy women is dominated by lactobacilli, which exerts important health-promoting effects to the host. In the present study, 261 lactobacilli isolated from vagina of healthy women were screened for their potential probiotic characteristics. Safety features (haemolytic activity, antibiotic susceptibility, bile salt hydrolase activity) and functional properties (resistance to low pH and bile salts, lysozyme tolerance, gastrointestinal survival, antagonistic activity against pathogens, hydrophobicity, auto-aggregation, and co-aggregation abilities, hydrogen peroxide production, biofilm formation, exopolysaccharide production, adhesion capacity to both normal human vagina epithelial cells and Caco-2 epithelial cells, and lactic acid production) were in depth evaluated. Seven strains, identified as *Lactobacillus rhamnosus*, *Lactobacillus helveticus* and *Lactobacillus salivarius* fulfilled the criteria described above. Therefore, the vaginal ecosystem represents a suitable source of probiotic candidates that could be used in new functional formulates for both gastrointestinal and vaginal eubiosis.

## Introduction

Probiotics are non-pathogenic, live microorganisms which, when administrated in adequate amounts, beneficially affect the health of the host^1^. During the last decade, researches in probiotic have progressed considerably and significant advances have been made in the selection and characterization of specific probiotic strains with health benefits^2^. The most studied probiotics belong to *Lactobacillus* and *Bifidobacterium* genera, which have been frequently associated with health-promoting effects in both humans and animals^3,4^. Previous studies have showed that probiotics improve immune system responses, stool consistency and vaginal lactobacilli density^5,6^. Since probiotic properties are highly strain-specific, every potential probiotic strain should be correctly identified, using both phenotypic and genotypic approaches. Moreover, each strain should be singularly investigated for the ability to survive throughout human gastrointestinal (GI) tract and to colonize a specific human tract^1^. Regarding safety feature, within species generally recognized as safe (GRAS), the FAO/WHO^1^ guidelines recommend to detect the antibiotic resistance pattern for each strain.

Comprehensive surveys of vaginal bacteria community have revealed that *Lactobacillus* species are dominant in the majority of healthy women, indicating this microenvironment as an excellent source of healthy lactobacilli. Notably, the beneficial effects of *Lactobacillus* spp. in vaginal ecosystem are based on a mutualistic relationship with other vaginal microbiota and human host^7^. It is interesting to highlight that more than 250 species of bacteria have already been detected by genomic sequencing in health vaginal environment^8^ and *Lactobacillus* species such as *Lactobacillus crispatus*, *Lactobacillus iners*, *Lactobacillus jensenii* and *Lactobacillus gasseri* are usually prevalent in women in reproductive age^9–12^. These species are able to produce several antimicrobial compounds (e.g. hydrogen peroxide, lactic acid) and to compete against pathogens for adhesion sites in the vaginal epithelium^13,14^, protecting from different diseases, including recurrent urinary infections, bacterial vaginosis, and vaginal candidiasis.

The main goal of the present study was to investigate the probiotic properties of lactobacilli isolated from healthy vaginal ecosystem in order to select promising lactobacilli strains to be use both as probiotic dietary supplements and food.

## Results

### Isolation and identification of vaginal lactobacilli

Three-hundred isolates were obtained from vaginal ecosystem of healthy Italian women and 261 of them were ascribed to *Lactobacillus* genus, based on phenotypic and genotypic identifications. As reported in Supplementary Fig. S1, species-specific PCR revealed that *Lactobacillus* isolates belonged to eight species: *L*. *gasseri* (28%), *L*. *salivarius* (20%), *L*. *crispatus* (18%), *L*. *helveticus* (13%), *L*. *fermentum* (10%), *L*. *rhamnosus* (10%), *L*. *paracasei* (1%) and *L*. *plantarum* (1%). In addition, the identity of the selected strains (P7, S7, P12, U13, E21, L3 and N30) was confirmed by the 16S rRNA gene sequencing and the accession number of each sequence was obtained.

### Hemolytic, BSH activity and antibiotic susceptibility

Among lactobacilli, none strain showed hemolytic activity. Regarding BSH activity, results revealed four *L*. *crispatus* (J36, U9, AB11, and AC7), three *L*. *gasseri* (A14, S21, Z9); one *L*. *helveticus* (P7) and one *L*. *salivarius* (N30) strains showed the ability to hydrolyse sodium salt of taurodeoxycholic acid (TDCA) (data not shown).

Variable susceptibility to antimicrobials was achieved, with the exception of *L*. *plantarum* and *L*. *paracasei* strains, which were sensitive to all antibiotics. Moreover, a high susceptibility was registered for tetracycline, erythromycin, and vancomycin, indicating a species and strain-dependent variability (Table 1).

**Table 1:** Antibiotic susceptibility measured by Etest method.

Species	%	TC	EM	VA	MZ^a^	NX^a^	TS^b^
*L*. *gasseri* (n = 72)	R	2	5	5	4	6	2
	S	70	67	67	68	66	70
*L*. *salivarius* (n = 52)	R	2	4	nr	12	3	4
	S	50	48	nr	40	49	48
*L*. *crispatus* (n = 47)	R	2	1	1	2	10	2
	S	45	46	46	45	37	45
*L*. *helveticus* (n = 33)	R	5	3	nr	7	1	1
	S	28	30	nr	26	32	32
*L*. *fermentum* (n = 26)	R	1	0	nr	2	1	10
	S	25	26	nr	24	25	16
*L*. *rhamnosus* (n = 25)	R	3	1	nr	0	0	0
	S	22	24	nr	25	25	25
*L*. *paracasei* (n = 3)	R	0	0	nr	0	0	0
	S	3	3	nr	3	3	3
*L*. *plantarum* (n = 3)	R	0	0	nr	0	0	0
	S	3	3	nr	3	3	3
% of resistance (R)		5.7	5.4	5.0	10.3	8.0	7.3
% of susceptibility (S)		94.3	94.6	95.0	89.7	92.0	92.7

Legend: R (resistant), S (susceptible), nr (not reqered) according to EFSA 2012.

TC, tetracycline; EM, erythromycin; VA, vancomycin; MZ, metronidazole; NX, norfloxacin; TS, trimethoprim/sulfamethoxazole.

^a^Etest max 256: the concentration on the strips was maximum 256 µg/ml.

^b^Etest max 32: the concentration on the strips was maximum 32 µg/ml.

In order to select lactobacilli to be used also in restoration therapy during antibiotic treatment, resistance to metronidazole (>256 μg/mL), norfloxacin (>256 μg/mL) and trimethoprim-sulfamethoxazole (>32 μg/mL) was detected. In detail, the highest resistance to metronidazole was observed in strains of *L*. *salivarius* (12/52) and *L*. *helveticus* (7/33); to norfloxacin in *L*. *gasseri* (6/72) and *L*. *crispatus* (10/47); to trimethoprim-sulfamethoxazole in strains belonging to *L*. *fermentum* (10/26).

### Acidic and bile salt tolerance

**Figure 1:**
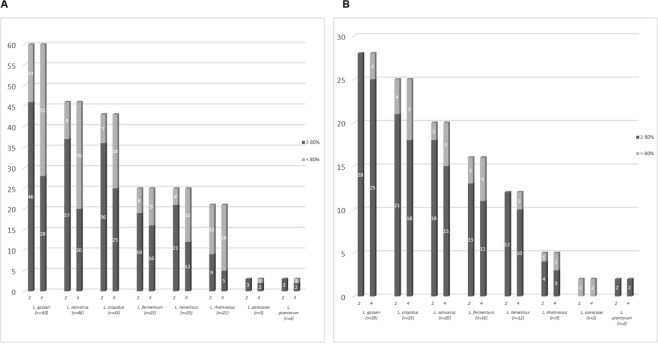
Survival rates of the Lactobacillus spp. strains at pH 3 after 2 and 4 hours of incubation (**A**) and at pH 2 after 2 and 4 hours of incubation (**B**).

Two hundred and twenty-six (226) *Lactobacillus* strains, selected as above, were screened for acidic tolerance. Starting from an initial number of viable cells (control cells) ranging from 9.0 to 9.5 log cfu/mL, a survival rates ≥80% were observed at both pH 3.0 and pH 2.0 (Fig. 1A,B). Results of tolerance to bile salts are shown in Supplementary Table S1. Overall, bile salts concentration of 0.5% (w/v) had no effect on most strains, with the exception of *L*. *crispatus* P10 and *L*. *plantarum* C11 and V7 strains, whereas at 1.0% (w/v) of bile salts the 86% and the 79% of the strains displayed bile tolerance after 2 and 4 h, respectively.

### Lysozyme tolerance

As reported in Supplementary Table S2, 13 strains belonged to *L*. *helveticus*, *L*. *rhamnosus*, and *L*. *salivarius* species, were categorized as lysozyme-resistant (survival rates ≥ 90%) after both 30 and 120 min (Supplementary Table S2); 43 strains as lysozyme-adaptive strains (survival rates 82–84%), and 10 strains, belonging to *L*. *gasseri*, *L*. *fermentum* and *L*. *salivarius* species, were grouped as lysozyme-sensitive (survival rates < 82%), according to Solieri *et al*.^15^. For these strains, a reduction of about 3 log unit and 4 log unit was observed after 30 and 120 min of exposition, respectively.

### Survival during *in vitro* GI transit

The lysozyme-adaptive and –resistant strains (56) were selected in order to evaluate their resistance during passage through the GI tract. Overall, 26 out of 56 strains showed the ability to survive during the GI transit, while 30 strains exhibited a strong reduction after exposure to gastric juice, registering a value of cell density approximately 5 log cfu/ml, which was maintained during pancreatic digestion (Supplementary Table S3).

### Antagonistic activity against pathogens

As showed in Table 2, the antagonistic activity of the selected 26 vaginal lactobacilli strains, against both GI and urogenital pathogens, was strain-dependent. Overall, strains belonging to *L*. *helveticus*, *L*. *rhamnosus* and *L*. *salivarius* species exhibited higher antagonistic activity (inhibition zone larger than 10 mm) against the tested pathogens than strains ascribed to *L*. *gasseri* and *L crispatus*. In particular, no inhibition zone vs most of the tested pathogens was registered by the latter species. it is interesting to point out that only 3 strains (F5, W18, E21) were able to inhibit *C*. *parapsilosis* and none displayed antagonistic activity vs *C*. *lusitaniae* (Table 2).

**Table 2:** Antimicrobial activity against gastrointestinal and urogenital pathogens.

Species	Strain	E. coli ATCC 25922	E. coli ATCC 700414	S. aureus ATCC 6538	L. monocytogenes DSM 12464	G. vaginalis ATCC 14018	C. albicans ATCC10231	C. krusei ATCC 14243	C. glabrata ATCC 90030	C. parapsilosis ATCC 90018	C. tropicalis ATCC 13803
L. crispatus (n = 4)	J31	−	−	−	−	−	−	−	−	−	−
	J36	+	−	−	−	−	−	−	−	−	−
	AB11	−	−	−	−	+	−	−	−	−	−
	AC7	+	−	−	−	−	−	−	−	−	−
L. gasseri (n = 6)	A9, A14	−	−	−	−	−	−	−	−	−	−
	A18	−	−	−	−	+	−	−	−	−	−
	F5	+	+	+	+	+	++	+	+	+	+
	W14	+	+	+	+	+	−	−	−	−	−
	W18	+	+	+	+	+	−	−	−	++	−
L. helveticus (n = 8)	C5	−	+	−	−	−	−	−	−	−	−
	P7	+++	+++	++	+++	+++	+++	++	++	−	++
	P12	++++	+++	+++	+++	+++	++	+++	++	−	++
	S7	+++	+++	++	++	++	++	++	++	−	++
	T5	−	+	−	−	−	−	−	−	−	−
	U13	++	++	++	+++	++	+++	+++	−	−	−
	Z3, Z4	+	−	−	−	−	−	−	−	−	−
L. rhamnosus (n = 3)	E21	+++	+++	++	++	+++	++	++	++	+	++
	L3	+++	+++	+++	++	+++	+++	+++	−	−	−
	L23	−	−	−	−	−	−	−	−	−	−
L. salivarius (n = 5)	H23, M23, Z15, AD12	−	−	−	−	−	−	−	−	−	−
	N30	++	++	++	+++	+++	++	+++	−	−	−

Legend: (−) no inhibition zone, (+) inhibition zone <10 mm; (++) inhibition zone 11–20 mm; (+++) inhibition zone >20 mm.

### Hydrophobicity, auto-aggregation, and co-aggregation abilities

Results of hydrophobicity, auto-aggregation, and co-aggregation detected for the vaginal lactobacilli are reported in Table 3. The cell surface hydrophobicity of the 10 selected strains ranged from 41 to 86%, with the exception of F5 and W18 strains, that displayed a value of 18% and 11%, respectively. *L*. *rhamnosus* E21 and L3 strains together with *L*. *helveticus* P7 and *L*. *salivarius* N30 showed the highest hydrophobicity (>70%) (Table 3). The auto-aggregation data ranged from 51% to 74%; only the F5, W14 and W18 strains showed value above 13%. The highest percentage was recorded by *L*. *helveticus* P12 strain, followed by the P7 strain. Overall, a broad range of variation in co-aggregation with pathogens was detected; seven strains (P7, S7, P12, U13, E21, L3, and N30) exhibited high co-aggregation with values higher than 50% (Table 3).

**Table 3:** Surface properties of the subset of 10 vaginal lactobacilli strains.

Species	Strains	H%	Auto-A%	CoA%			
				*E*. *coli 555*	*G*. *vaginalis*	*C*. *albicans*	*C*. *glabrata*
*L*. *gasseri*	F5	18.12 ± 0.07^b^	12.23 ± 0.09^c^	14.18 ± 0.12^c^	15.23 ± 0.14^b^	24.33 ± 0.14^c^	6.13 ± 0.18^a^
	W14	41.08 ± 0.13^c^	7.05 ± 0.20^b^	12.23 ± 0.18^b^	18.21 ± 0.17^c^	11.21 ± 0.16^b^	8.06 ± 0.11^b^
	W18	11.15 ± 0.07^a^	6.21 ± 0.09^a^	6.35 ± 0.04^a^	14.38 ± 0.11^a^	9.37 ± 0.09^a^	12.54 ± 0.21^c^
*L*. *helveticus*	P7	73.21 ± 0.09^g^	71.24 ± 0.06^i^	59.25 ± 0.11^g^	58.23 ± 0.28^e^	67.34 ± 0.12^h^	71.28 ± 0.23^i^
	S7	46.30 ± 0.16^e^	57.43 ± 0.16^f^	51.25 ± 0.09^d^	60.31 ± 0.21^f^	52.28 ± 0.19^d^	54.67 ± 0.17^d^
	P12	48.26 ± 0.04^f^	74.33 ± 0.07^l^	68.22 ± 0.10^i^	72.37 ± 0.11^l^	58.23 ± 0.17^e^	63.47 ± 0.21^g^
	U13	42.34 ± 0.09^d^	51.15 ± 0.10^d^	54.25 ± 0.12^f^	67.29 ± 0.18^h^	52.43 ± 0.12^d^	55.28 ± 0.28^e^
*L*. *rhamnosus*	E21	82.12 ± 0.09^i^	61.26 ± 0.04^g^	60.31 ± 0.07^h^	66.27 ± 0.15^g^	63.27 ± 0.15^g^	68.27 ± 0.09^h^
	L3	86.18 ± 0.10^l^	55.27 ± 0.09^e^	53.28 ± 0.16^e^	57.23 ± 0.16^d^	72.28 ± 0.18^i^	58.23 ± 0.11^f^
*L*. *salivarius*	N30	76.23 ± 0.10^h^	66.32 ± 0.16^h^	71.51 ± 0.11^l^	71.28 ± 0.14^i^	61.38 ± 0.11^f^	75.34 ± 0.26^l^

Legend: H%: Hydrophobicity; Auto-A%: auto-aggregation; CoA%: co-aggregation.

Results are expressed as average value and standard deviation of three separate experiments. Different letters (a–l) in the same column indicate significant differences by One-way ANOVA test, followed by Tukey post-hoc test (p < 0.05).

### Hydrogen peroxide, exopolysaccharides, lactic acid production and biofilm formation

Results of hydrogen peroxide (H_2_O_2_) production are reported in Table 4. The qualitative analysis demonstrated that all the 10 selected strains produced H_2_O_2_. In particular, *L*. *helveticus* P7 and *L*. *rhamnosus* E21 and L3 strains showed high H_2_O_2_ production, while *L*. *salivarius* (N30) and *L*. *helveticus* (S7, P12, U13) strains recorded moderate H_2_O_2_ production. All *L*. *gasseri* strains exhibited low ability to produce H_2_O_2_.

**Table 4:** Hydrogen peroxide, biofilm, exopolysaccharides, L- and D-lactic acid production abilities of the tested vaginal lactobacilli strains.

Species	Strains	H2O2*	Biofilm**	EPS (mg/l)	L-lactic acid (mmol/l)	D-lactic acid (mmol/l)
*L*. *gasseri*	F5	1	Moderate	153 ± 1.2^c^	2.28 ± 0.13^a^	5.12 ± 0.14^a^
	W14	1	NB	104 ± 1.6^a^	2.13 ± 0.09^a^	4.74 ± 0.14^a^
	W18	1	NB	138 ± 2.1^b^	2.09 ± 0.17^a^	6.65 ± 0.14^b^
*L*. *helveticus*	P7	3	Very strong	196 ± 2.1^d^	6.91 ± 0.17^c^	13.11 ± 0.22^c^
	S7	2	Moderate	191 ± 2.4^d^	5.74 ± 0.14^b^	12.81 ± 0.09^c^
	P12	2	Very strong	236 ± 0.5^g^	7.03 ± 0.12^c^	13.04 ± 0.20^c^
	U13	2	Moderate	202 ± 1.2^d^	5.64 ± 0.31^b^	12.72 ± 0.17^c^
*L*. *rhamnosus*	E21	3	Moderate	212 ± 1.2^e^	5.67 ± 0.29^b^	12.91 ± 0.14^c^
	L3	3	Moderate	228 ± 1.6^f^	7.71 ± 0.21^d^	12.88 ± 0.18^c^
*L*. *salivarius*	N30	2	Strong	268 ± 2.9^h^	8.94 ± 0.13^e^	12.94 ± 0.11^c^
*L*. *acidophilus*	ATCC 4356	3	nt	nt	7.78 ± 0.12^d^	12.76 ± 0.15^c^

Legend: *The strains were scored as 1 (low producer, time >20 min), 2 (medium producer, time 10–20 min) and 3 (high producer, time <10 min). **The strains were classified as non-biofilm (NB) producers (OD ≤ ODc); weak biofilm producers (ODc < OD ≤ 2 × ODc); moderate biofilm producers (2moderate biofilm producers ODc < OD ≤ 4 × ODc); strong biofilm producers (4 × ODc < OD ≤ 8 × ODc) and very strong biofilm producers (8 × ODc < OD).

Different letters (a–h) in the same column indicate significant differences by One-way ANOVA test, followed by Tukey post-hoc test (p < 0.05). nt: not tested.

All lactobacilli were able to produce biofilm, with the exception of *L*. *gasseri* W14 and W18 strains (Table 4). In addition, all strains were able to produce EPS with values ranging from 104 mg/L to 268 mg/L; *L*. *salivarius* N30 (268 mg/L) and *L*. *helveticus* P12 (236 mg/L) produced the highest amount of EPS. Table 4 also shown the concentration of lactic acids (L and D) produced by the 10 vaginal lactobacilli, ranging from 2.09 mmol/l to 8.94 mmol/l and 4.74 mmol/l to 13.11 mmol/l, for L- lactic and D-lactic acids, respectively.

### *In vitro* adhesion assay

**Figure 2:**
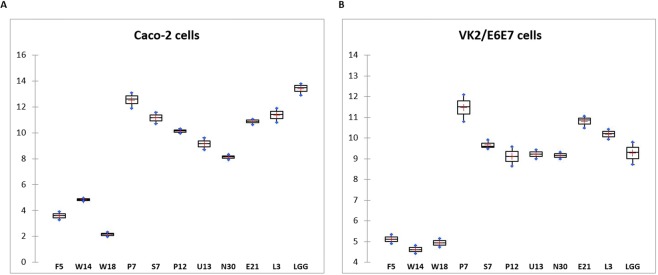
Adhesion (%) of lactobacilli to Caco-2 and to VK2/E6E7 vaginal epithelial cells.

The adhesion ability of the 10 selected *Lactobacillus* strains to Caco-2 and to VK2/E6E7 vaginal epithelial cells, in comparison with the reference probiotic strain Lactobacillus *rhamnosus* GG, is shown in Fig. 2. Overall, the adhesion capacity was strain-dependent. *L*. *helveticus* P7 and *L*. *rhamnosus* E21 and L3 strains exhibited the highest binding ability to both Caco-2 and VK/E6E7 cells.

## Discussion

Over the past few decades, the probiotics ability to exert health benefits has prompted increased both scientific interest and industry demand for food and supplement. Several studies have been carried out on beneficial effects exert by probiotics and it is already well demonstrated that functional properties are strain-dependent. The healthy vaginal ecosystem is dominated mainly by *Lactobacillus* spp.^16^, suggesting it as suitable source of isolation.

In the present study, 261 lactobacilli isolated from vaginal microbiota of Italian healthy women were screened for their potential probiotic characteristics. According to other studies, the lactobacilli isolates were mainly assigned, through molecular identification, to *L*. *gasseri*, *L*. *salivarius* and *L*. *crispatus* species, widely recognized as indicator of healthy vaginal microbiota^17–19^. Although *Lactobacillus*-dominated vaginal microbiota include the *L*. *iners* species^11,20^, the culture –dependent approach used did not reveal isolates belonging to this species. This could be due to its stringent nutritional requirements and very low oxygen tolerance, in accordance to Parolin *et al*.^21^. *L*. *iners* dominated vaginal community type seems to be less stable or more in transition than the other community types and more associated with vaginal dysbiosis, since it has clonal variants that in some cases promote a healthy vagina, and in other cases are associated with dysbiosis and disease^22,23^. In addition, although differences between ethnic groups is still not clear, *L iners* has been more often detected in Black African and Afro-American women compared to Caucasian or Asian women^24–27^.

Albeit lactobacilli have a long history of safe use, to be qualified as probiotic, safety properties should be firstly addressed^28^. Our tested strains appeared to be safe, since none caused the lysis of erythrocytes of sheep blood. In addition, to prevent the transfer of resistance to endogenous bacteria, probiotic should not carry any resistance^29^. Regarding the antimicrobial susceptibility, the phenotypical resistance to several antibiotics was performed according to international standards and guidelines^30^. Results confirmed that the majority of the strains was sensitive to most of the tested antibiotics, even if a strains-dependent profile was revealed^31–35^. Even if no data on resistance genes nor on the cellular localization of them was made in the present study, our results are in agreement with previous works for a broad range of antibiotics, although different nutrient media, incubation conditions and/or susceptibility testing methods were used^35–39^. Focusing on the phenotypical vancomycin resistance, it is noteworthy that most of the lactobacilli species are intrinsically resistant to this antibiotic, which is attributed to the synthesis of modified cell wall peptidoglycan precursors^40,41^. This type of resistance does not represent a concern for a probiotic, as it is different from the inducible, transferable mechanism observed in other bacteria, such as enterococci^42^. Our results revealed that only few strains (6 out of 119), belonging to *L*. *crispatus* and *L*. *gassseri* species, exhibited the phenotypical resistance to vancomycin, confirming the high susceptibility to this antibiotic of the *L*. *acidophilus* group^43^. Similarly, to some other reports^35,44^, high level of resistance to norfloxacin, metronidazole, and trimethoprim-sulfamethoxazole was also revealed. In detail, resistance to metronidazole and to sulphonamides, antimicrobials generally used for the treatment of bacterial vaginosis, trichomoniasis and urinary tract infections, is considered a positive feature for selection of probiotic to be used in supporting vaginal microbiota restoration therapy^31,45–47^. Beyond safety properties, an important step towards the selection of probiotic strains is the ability to survive the passage through the GI tract. It is interesting to highlight that, in the present study, strains belonging to *L*. *rhamnosus* (E21, and L3), *L*. *helveticus* (P7, P12, S7, and U13), and *L*. *salivarius* (N30) species fulfilled all probiotic selection criteria, exhibiting high survival during *in vitro* GI passage, adhesion to both intestinal and vaginal epithelia, hydrophobicity, auto-aggregation, and co-aggregation, in accordance to previous studies^48,49^. The co-aggregation is an important property of lactobacilli, because it can create a microenvironment around the pathogens with a high concentration of inhibitory substances, preventing pathogens adhesion to intestinal and/or vaginal epithelium^47^. In this regard, the aforementioned strains showed antagonistic activity against the majority of the pathogens, including *Candida* spp., in accordance to previous study^21,50^, corroborating the useful application of lactobacilli in the prevention and treatment of candidiasis. This activity is mainly attributed to the production of antimicrobial substances or metabolites such as organic acids (e.g lactic acid) and hydrogen peroxide^50^. In our study, higher amounts of these compounds were produced by lactobacilli strains ascribed to *L*. *rhamnosus*, and *L*. *salivarius* and *L*. *helveticus*, species. *L*. *helveticus* is not generally recognized as a dominant species in the vaginal ecosystem, and its presence need to be clarify, since it is a resilient microorganism of the human GI tract^51^. Recently, Pino *et al*.^52^ revealed, for the first time, the dominance of *L*. *helveticus* in the vaginal ecosystem of Italian women treated with lactoferrin, confirming its transient condition from faecal human. *L*. *helveticus* is a GRAS species, which received the QPS status by the European Food Safety Authority (EFSA). It is generally used as thermophilic starter in dairy fermentation, and it is a dominant species found in several Italian cheeses^53,54^. Besides its technological importance, many scientific evidences showed that strains belonging to the *L*. *helveticus* species have health-promoting properties^55^ due to the ability to stimulate the immune system, to defence the host against pathogens, to influence the intestinal microbiota composition^56,57^. Despite *L*. *crispatus* is considered one of the most active species that contributes to the maintenance of normal vaginal microbiota and its absence has been associated with a range of vaginal abnormalities^23,58,59^, in the present study none of the *L*. *crispatus* strains was included among the selected promising probiotic. This aspect could be explained taking into account that probiotic properties are strain-dependent and not species-dependent.

In conclusion, the present study demonstrates that vaginal ecosystem is an excellent source of promising *L*. *helveticus* probiotic strains, which could be proposed as indicator of healthy vaginal status and used both in new functional supplements and food.

## Methods

### Reference strains and culture conditions

The bile salt hydrolase (BSH)-positive strain *Lactobacillus acidophilus* DRU, the hydrogen peroxide (H_2_O_2_) producer *Lactobacillus acidophilus* ATCC 4356, and the reference strain *Lactobacillus rhamnosus* GG (ATCC 53103) were routinely cultured in de Man Rogosa and Sharpe (MRS, Biolife, Italy) medium plus 100 mg/L of cycloheximide (Merck, Germany) at 37 °C under anaerobic conditions, using Anaerocult C (Merck, Milan, Italy). The haemolytic positive strains *Streptococcus pyogenes* ATCC 19615 and *Streptococcus pneumoniae* ATCC 6303 were cultured on Brain-Heart Infusion (BHI, Becton Dickinson GmbH, Germany) at 37 °C under 5% CO_2_ conditions. *Escherichia coli* 555, *E*. *coli* ATCC 25922, *E*. *coli* ATCC 700414, and *Staphylococcus aureus* ATCC 6538 were routinely cultured on Trypticase Soy Broth medium (Oxoid, Milan) at 37 °C, under aerobic conditions. *Listeria monocytogenes* DSM 12464 strain was reactivated in BHI broth at 30 °C. *Gardnerella vaginalis* ATCC 14018 was cultured on Casman’s medium base added of 5% of rabbit blood (VWR, Milan, Italy) at 37 °C. *Candida albicans* ATCC 10231, *Candida krusei* ATCC 14243, *Candida glabrata* ATCC 90030, *Candida parapsilosis* ATCC 90018, *Candida lusitaniae* ATCC 200951 and *Candida tropicalis* ATCC 13803 were cultured on Yeast Mold Broth (Conda, Madrid, Spain) at 28 °C in aerobic conditions.

### Sampling and isolation of lactobacilli

Lactobacilli were isolated from vaginal ecosystem of asymptomatic Italian women, which were invited to participate in the study during their routine gynecological consultations. Thirty participants aged between 18 and 36 years, with regular menstrual cycles and with healthy vaginal mucosa and vaginal cytology for cancer presenting normal findings, were recruited at Obstetrics and Gynecology Department, General Hospital G. Rodolico, Catania, Italy, between September 2017 and February 2018. The exclusion criteria were: antibiotic, probiotic, immune suppressants or exogenous hormone treatments; neoplasia in the genital area; pregnancy or breastfeeding; neurological and/or psychiatric disorders; clinically apparent herpes simplex infection or defined diagnosed human papillomavirus, herpes simplex virus type 2, or human immunodeficiency virus type 1 infection; *Chlamydia*, yeasts, *Neisseria gonorrhoeae*, *Trichomonas vaginalis* infection and bacterial vaginosis (BV). Medical history concerning contraceptive use, infectious disease history, sexual activity, and last menstrual period were assessed at recruitment and demographic characteristics of participating women are reported in Supplementary Table S4. Sampling procedures were carried out following ethical standards of the responsible committee on human experimentation (institutional and national) and according to the Helsinki Declaration of 1975, as revised in 2008. The study protocol was approved by the local ethics committee (registration number SHI-EVE-2014.01). Informed consent was obtained from all study participants before they were enrolled. Vaginal discharge samples were collected and analyzed as previously described^40^. Rogosa Bios Agar (Biolife, Italy), MRS and BHI agar plates were used. Both MRS and BHI agar plates were supplemented with 0.05% of L-cysteine and anaerobically incubated at 37 °C for 24–48 h, using Anaerocult C (Merck, Milan, Italy). Individual colonies were randomly selected, purified, tested for catalase activity and Gram reaction, and microscopically examined before storing at −80 °C in liquid culture, using 20% of glycerol.

### Identification of lactobacilli

Lactobacilli isolates were genotypically identified based on the 16S rRNA gene analysis. Total genomic DNA was extracted following the method previously described^60^. DNA concentration and purity were determined using the NanoDrop 2000, (Thermo Fisher Scientific, USA). DNA integrity and size were checked by 1.0% agarose gel electrophoresis containing GelRed Nucleic Acid Gel Stain (Biotium, Italy). *Lactobacillus* isolates were identified at genus level using the primer pairs LbLMA1-rev and R16-1, as suggested by Dubernet *et al*.^61^. Isolates exhibiting amplification products were subjected to species-specific PCR, using primer pairs and conditions reported in Supplementary Table S5. PCR reactions were carried out in a final volume of 50 μL, containing 25 ng of template DNA, 2.5 U of Taq DNA polymerase (Invitrogen, Italy), 10 mM Tris-HCl (pH 8.4), 50 mM KCl, 1.5 mM MgCl_2_, 200 μM of each dNTPs, and 10 pmol of each primer. The PCR products were resolved by electrophoresis using a 1.0% agarose gel in 1X TBE buffer (89 mM Tris–borate, 89 mM boric acid, 2 mM EDTA; pH 8.0) and visualized after staining with Gel Red Nucleic Acid Stain.

### Safety assessment

#### Haemolytic activity

*Lactobacillus* strains, grown in MRS broth for 18–24 h at 37 °C, were streaked onto blood agar plates containing sheep blood (Biolife, Milan, Italy), and incubated, under anaerobic conditions, at 37 °C for 24–48 h. The haemolytic activity was visually detected and distinguished as β-haemolysis, α-haemolysis, or γ-haemolysis based on the appearance of a clear zone, green halo or no zones around colonies, respectively. *S*. *pyogenes* ATCC 19615 and *S*. *pneumoniae* ATCC 6303 were used as positive controls.

#### Antibiotic susceptibility and MIC determination

The strains were considered antibiotic resistant or sensitive, according to breakpoints proposed by European Food Safety Authority^30^. In addition, for the four antimicrobials (metronidazole, nitrofurantoin, norfloxacin, trimethoprim/sulfamethoxazole), not included in the EFSA list, resistances were determined in accordance to Štšepetova *et al*.^31^. The minimum inhibitory concentration (MIC) was determined by the Etest® method (BioMérieux, Marcy l′Etoile, France), using the LAB susceptibility test medium (LSM) agar formulation, as recommended by ISO 10932/IDF 223^62^.

#### Bile salt hydrolase activity

Bile salt hydrolase (BSH) activity was determined following the method previously reported by Caggia and co-workers^63^. The appearance of a precipitate around colony was considered as a positive sign and, based on the confluence of the precipitate, each strain was coded as ‘+++’ for heavy; ‘++’ for intermediate; ‘+’ for low; and ‘−’ for no precipitation. The strain *L*. *acidophilus* DRU was used as positive control.

### Functional properties

#### Resistance to acidic conditions and bile salts

The acidic tolerance of the vaginal lactobacilli was detected on MRS at pH 2.0 and 3.0, obtained by 1 M HCl adding. MRS at pH 6.2 was used as control. Lactobacilli were cultured twice in MRS broth and bacterial suspension (10^9^ cfu/mL) was inoculated into acidified medium. Aliquots were taken immediately after inoculation (0 h), and after 2 and 4 h of incubation at 37 °C. Acidic resistance was determined as survival rate percentage (SR %), based on initial and final number of viable cells enumerated on MRS agar after 48 h. Lactobacilli strains showing survival rate higher than 80%, after 4 h of incubation, were further tested for bile salts tolerance. In detail, bovine bile salts (Oxgall; Sigma-Aldrich), at final concentrations of 0.5% and 1.0%, were added to MRS broth. Medium without bovine bile salts was used as control. The strains were inoculated at final cell density of 10^9^ cfu/mL and anaerobically incubated at 37 °C up to 4 h. The survival rates were determined, after 2 and 4 h, as described before.

#### Lysozyme tolerance

The tolerance of the selected strains to lysozyme was evaluated as previously described^63,64^. Aliquots, withdrawn at 0, 30 and 120 min, were opportunely diluted and viable bacteria (cfu/mL) were enumerated by plating on MRS agar. Bacterial suspension in sterile electrolyte solution without lysozyme was used as control.

#### Survival during gastrointestinal transit

The lactobacilli’s ability to survive during the gastrointestinal (GI) transit was *in vitro* determined on simulated gastric juice (SGJ) and on simulated intestinal fluid (SIF), as described by Pithva *et al*.^65^ with slight modifications. In details, SGJ (0.3% pepsin, 0.5% NaCl, adjusted to pH 2 by adding 1 M HCl) and SIF (0.1% pancreatin, 0.5% bile salt, 0.5% NaCl, 0.4% phenol, adjusted to pH 8 by adding 1 M NaOH) were prepared immediately before use and sterilized using 0.22 µm cellulose acetate filter (Minisart filters, Sartorius, Goettingen, Germany). All chemicals were obtained from Sigma Aldrich (St. Louis, MO). Bacterial cells, from overnight cultures, were harvested by centrifugation and re-suspended in Phosphate Buffer solution (PBS), to obtain a 10^9^ cells/mL bacterial suspension. The obtained cell suspension was mixed with SGJ and incubated for 2 h at 37 °C, in microaerophilic conditions under agitation (200 rpm). The cells, pelleted by centrifugation, were re-suspended in SIF and incubated at 37 °C for 3 h. SGJ and SGJ–SIF-treated cells were serially diluted, and plated on MRS agar for the determination of cell viability.

#### Antagonistic activity against pathogens

Lactobacilli were tested for antagonistic activity using *E*. *coli* ATCC 700414, *E*. *coli* ATCC 25922, *S*. *aureus* ATCC 6538, *L*. *monocytogenes* DSM 12464, *G*. *vaginalis* ATCC 14018, *C*. *albicans* ATCC 10231, *C*. *krusei* ATCC 14243, *C*. *glabrata* ATCC 90030, *C*. *parapsilosis* ATCC 90018, *C*. *lusitaniae* ATCC 200951 and *C*. *tropicalis* ATCC 13803 as target bacteria. The assay was performed by the agar spot test. After incubation for 48 h, the appearance of inhibition zones around lactobacilli spots were visually detected and, based on diameter sizes, results were expressed as: (−) no inhibition zone; (+) inhibition zone < 10 mm; (++)inhibition zone between 11 and 20 mm; (+++) inhibition zone >20 mm.

#### Hydrophobicity, auto-aggregation, and co-aggregation abilities

Vaginal lactobacilli strains were subjected to cell surface hydrophobicity (H%) assay as described by Caggia *et al*.^63^. The auto-aggregation (Auto-A%) and co-aggregation (Co%) abilities were tested according to Solieri *et al*.^15^. In co-aggregation assay *E*. *coli* 555, *G*. *vaginalis* ATCC 14018, *C*. *albicans* ATCC10231, and *C*. *glabrata* ATCC 90030 were used as pathogenic strains.

#### Hydrogen peroxide production

The ability to produce hydrogen peroxide (H_2_O_2_) was evaluated by culturing the strains on MRS agar containing 0.25 mg/mL of 3,3′,5,5′-tetramethylbenzidine and 0.01 mg/mL of horseradish peroxidase, in anaerobic conditions, for 72 h. The plates were air exposed and the H_2_O_2_ production was evaluated based on the time required for a blue coloration appearance. The tested strains were scored as low (score 1, time >20 min), medium (score 2, time 10–20 min) and high (score 3, time <10 min) H_2_O_2_ producer. Strains not producing the blue coloration were scored as 0. *L*. *acidophilus* ATCC 4356 was used as positive control.

#### Biofilm formation

The ability of the vaginal strains to develop biofilm was evaluated according to Pérez Ibarreche *et al*.^66^. The optical density (OD) at 570 nm, of each well was measured using a microplate reader (iMark Microplate Reader, Biorad). MRS medium without inoculum was included as negative control. A cut-off OD (ODc) criterion was considered based on three standard deviations above the OD mean value registered for the negative control. The strains were considered non-biofilm producers (OD ≤ ODc); weak biofilm producers (ODc < OD ≤ 2 × ODc); moderate biofilm producers (2 x ODc < OD ≤ 4 × ODc); strong biofilm producers (4 × ODc < OD ≤ 8 × ODc) and very strong biofilm producers (8 × ODc < OD)^67^.

#### Exopolysaccharide production

The exopolysaccharide production was quantitatively estimated by using the phenol/sulphuric acid method^68^. The amount of total exopolysaccharide (expressed as mg/L) was estimated using glucose (50–500 mg/L) as standard^69^.

#### *In vitro* adhesion assay

The screened lactobacilli strains were studied for adhesion capacity using both normal human vagina epithelial cells (VK2/E6E7 ATCC-CRL-2616) and Caco-2 epithelial cells (ATCC HTB-37) according to Petrova *et al*.^48^. The adhesion ability, expressed as percentage, was calculated comparing the number of adherent cells to the initial viable count of the added bacterial suspension (10^7^ cfu/ml).

#### Lactic acid production

Type and concentration of lactic acid produced by lactobacilli strains were determined on cell free culture supernatant using the L (+) and D (−) lactate dehydrogenase kit (Megazyme International Ireland Ltd., Co. Wicklow, Ireland), following the manufacture’s instruction. The assays were specific for both D-lactic acid and L-lactic acid.

### Statistical analysis

All data were expressed as a mean and standard deviation of triplicate independent experiments. Significant ANOVA results were followed up with Tukey’s Multiple Comparison Test and differences were considered statically significant when p < 0.05.

### Nucleotide sequence accession numbers

The sequences of the 16S rDNA of the 7 strains, selected based on the characteristics mentioned above, were deposited in the GenBank database. The accession numbers of the strains are as follows (isolates code in parentheses): MK389414 (P7), MK389415 (S7), MK389416 (P12), MK389417 (U13), MK389418 (E21), MK389419 (L3), MK389420 (N30).

## Supplementary information

supplementary information
