# Supplementary material for: Detection of vaginal lactobacilli as probiotic candidates
Source: Sci Rep. 2019 Mar 4;9:3355. doi: 10.1038/s41598-019-40304-3 (PMC6399336; doi:10.1038/s41598-019-40304-3)
Supplement: Supplementary file 1 — supplementary information [file 41598_2019_40304_MOESM1_ESM.docx]

**Supplementary information**

**Detection of vaginal lactobacilli as probiotic candidates**

Alessandra Pino^1^, Emanuela Bartolo^2^, Cinzia Caggia^1^, Antonio Cianci^2^, Cinzia L. Randazzo^1*^

^1^Department of Agricultural, Food and Environment, University of Catania, CT, IT

^2^Department of General Surgery and Medical Surgical Specialties, Gynecological Clinic, University of Catania, Policlinico Universitario, Catania, Italy.

***** Corresponding Author: [crandra@unict.it](mailto:crandra@unict.it)

**Supplementary Figure S1** Species assignment of lactobacilli isolated from vaginal swabs.

**Supplementary Table S1** Survival rate (SR %) of *Lactobacillus* strains after 2 and 4 hours in MRS containing 0.5% and 1.0 % of bile salts.

| Species | Strain | Bile salts | | | |
| --- | --- | --- | --- | --- | --- |
|  |  | 0.5 % | | 1.0 % | |
|  |  | SR% 2h | SR% 4h | SR% 2h | SR% 4h |
| *L. gasseri* (n=25) | A4 | 96 | 80 | 94 | 81 |
|  | A7 | 95 | 86 | 94 | 80 |
|  | A8 | 94 | 82 | 92 | 80 |
|  | A9 | 95 | 83 | 92 | 82 |
|  | A14 | 88 | 81 | 88 | 82 |
|  | A18 | 88 | 80 | 91 | 80 |
|  | B3 | 93 | 81 | 89 | 77 |
|  | B5 | 89 | 83 | 88 | 77 |
|  | F5 | 96 | 86 | 92 | 82 |
|  | G12 | 91 | 83 | 88 | 78 |
|  | H7 | 91 | 84 | 89 | 80 |
|  | K2 | 90 | 86 | 89 | 86 |
|  | K7 | 97 | 83 | 95 | 81 |
|  | M9 | 95 | 82 | 91 | 81 |
|  | P5 | 91 | 82 | 90 | 83 |
|  | S21 | 90 | 80 | 89 | 80 |
|  | T13 | 90 | 84 | 89 | 81 |
|  | U3 | 93 | 82 | 91 | 80 |
|  | U5 | 93 | 81 | 91 | 80 |
|  | W14 | 91 | 84 | 89 | 82 |
|  | W18 | 91 | 83 | 90 | 82 |
|  | Y13 | 92 | 84 | 91 | 82 |
|  | Z9 | 92 | 83 | 91 | 81 |
|  | AB18 | 92 | 80 | 89 | 77 |
|  | AC3 | 89 | 81 | 89 | 80 |
| *L. crispatus* (n=18) | C9 | 98 | 89 | 89 | 84 |
|  | D18 | 94 | 83 | 91 | 85 |
|  | D27 | 93 | 80 | 87 | 83 |
|  | E3 | 88 | 81 | 84 | 82 |
|  | G8 | 81 | 80 | 83 | 82 |
|  | J16 | 85 | 80 | 71 | 53 |
|  | J31 | 95 | 89 | 89 | 87 |
|  | J36 | 96 | 91 | 84 | 81 |
|  | N3 | 98 | 92 | 97 | 91 |
|  | P10 | 90 | 79 | 73 | 59 |
|  | S18 | 87 | 85 | 92 | 88 |
|  | T4 | 86 | 82 | 90 | 87 |
|  | U9 | 86 | 81 | 88 | 87 |
|  | V26 | 88 | 82 | 89 | 88 |
|  | Y11 | 86 | 83 | 88 | 83 |
|  | AB11 | 85 | 80 | 84 | 83 |
|  | AC7 | 82 | 80 | 84 | 82 |
|  | AC8 | 81 | 80 | 74 | 73 |
| *L. salivarius* (n=15) | C21 | 91 | 82 | 88 | 80 |
|  | D20 | 91 | 83 | 88 | 80 |
|  | G18 | 91 | 84 | 89 | 82 |
|  | H23 | 92 | 83 | 91 | 82 |
|  | J23 | 94 | 81 | 92 | 80 |
|  | L21 | 93 | 80 | 91 | 77 |
|  | M23 | 91 | 82 | 91 | 81 |
|  | N11 | 91 | 86 | 78 | 75 |
|  | N30 | 90 | 82 | 90 | 80 |
|  | O29 | 91 | 80 | 90 | 80 |
|  | P35 | 91 | 80 | 89 | 80 |
|  | T14 | 90 | 82 | 90 | 82 |
|  | Y21 | 90 | 82 | 77 | 70 |
|  | Z15 | 91 | 80 | 88 | 80 |
|  | AD12 | 91 | 80 | 90 | 80 |
| *L. fermentum* (n=11) | E11 | 98 | 80 | 96 | 80 |
|  | E18 | 95 | 86 | 95 | 84 |
|  | O13 | 88 | 82 | 88 | 83 |
|  | O16 | 94 | 86 | 87 | 82 |
|  | W4 | 86 | 80 | 73 | 65 |
|  | W17 | 84 | 80 | 68 | 67 |
|  | X10 | 80 | 80 | 66 | 57 |
|  | X13 | 80 | 80 | 68 | 67 |
|  | AB2 | 96 | 87 | 89 | 84 |
|  | AC5 | 90 | 81 | 80 | 73 |
|  | AD1 | 91 | 85 | 78 | 68 |
| *L. helveticus* (n= 10) | C5 | 95 | 85 | 93 | 83 |
|  | G7 | 95 | 83 | 89 | 80 |
|  | G13 | 94 | 88 | 90 | 82 |
|  | P7 | 88 | 84 | 87 | 83 |
|  | P12 | 88 | 81 | 85 | 81 |
|  | S7 | 91 | 80 | 88 | 81 |
|  | T5 | 90 | 81 | 88 | 80 |
|  | U13 | 92 | 86 | 89 | 83 |
|  | Z3 | 91 | 86 | 90 | 85 |
|  | Z4 | 93 | 84 | 91 | 82 |
| *L. rhamnosus* (n=3) | E21 | 93 | 82 | 90 | 80 |
|  | L3 | 95 | 84 | 91 | 81 |
|  | L23 | 91 | 85 | 92 | 85 |
| *L. plantarum* (n=2) | C11 | 80 | 69 | 69 | 64 |
|  | V7 | 84 | 70 | 71 | 66 |

Data are reported as mean survival rate (SR%) of three independent experiments. SR% was calculated as following: viable cells in MRS (0.5% or 1.0% of bile salts)/viable cells in control (without bile salt) × 100.

**Supplementary Table S2** Lysozyme tolerance, expressed as survival rate (SR %) of the tested vaginal lactobacilli strains.

| Species | Strain | Lysozyme tolerance | |
| --- | --- | --- | --- |
|  |  | SR% 30 min | SR% 120 min |
| *L. gasseri* (n=21) | A4 | 76 | 58 |
|  | A7 | 73 | 58 |
|  | A8 | 69 | 59 |
|  | A9 | 84 | 85 |
|  | A14 | 86 | 88 |
|  | A18 | 84 | 87 |
|  | F5 | 82 | 85 |
|  | H7 | 73 | 59 |
|  | K2 | 85 | 87 |
|  | K7 | 73 | 61 |
|  | M9 | 84 | 86 |
|  | P5 | 85 | 86 |
|  | S21 | 84 | 86 |
|  | T13 | 83 | 85 |
|  | U3 | 72 | 59 |
|  | U5 | 84 | 85 |
|  | W14 | 84 | 85 |
|  | W18 | 83 | 84 |
|  | Y13 | 84 | 86 |
|  | Z9 | 83 | 84 |
|  | AC3 | 83 | 85 |
| *L. crispatus* (n=15) | C9 | 84 | 85 |
|  | D18 | 84 | 86 |
|  | D27 | 83 | 88 |
|  | E3 | 83 | 84 |
|  | G8 | 83 | 84 |
|  | J31 | 84 | 86 |
|  | J36 | 84 | 85 |
|  | N3 | 83 | 84 |
|  | S18 | 84 | 85 |
|  | T4 | 83 | 85 |
|  | U9 | 84 | 86 |
|  | V26 | 84 | 86 |
|  | Y11 | 83 | 85 |
|  | AB11 | 83 | 84 |
|  | AC7 | 84 | 85 |
| *L. salivarius* (n=12) | C21 | 84 | 85 |
|  | D20 | 94 | 91 |
|  | G18 | 84 | 85 |
|  | H23 | 84 | 86 |
|  | J23 | 68 | 60 |
|  | M23 | 84 | 86 |
|  | N30 | 95 | 92 |
|  | O29 | 97 | 92 |
|  | P35 | 84 | 85 |
|  | T14 | 84 | 85 |
|  | Z15 | 96 | 93 |
|  | AD12 | 84 | 87 |
| *L. helveticus* (n=10) | C5 | 84 | 85 |
|  | G7 | 83 | 86 |
|  | G13 | 84 | 85 |
|  | P7 | 98 | 95 |
|  | P12 | 96 | 95 |
|  | S7 | 96 | 91 |
|  | T5 | 84 | 87 |
|  | U13 | 99 | 96 |
|  | Z3 | 96 | 97 |
|  | Z4 | 96 | 93 |
| *L. fermentum* (n=5) | E11 | 64 | 73 |
|  | E18 | 83 | 84 |
|  | O13 | 67 | 61 |
|  | O16 | 73 | 66 |
|  | AB2 | 81 | 83 |
| *L. rhamnosus* (n=3) | E21 | 93 | 90 |
|  | L3 | 95 | 92 |
|  | L23 | 95 | 93 |

Data are reported as mean survival rate (SR%) of three independent experiments. SR% was calculated as following: viable cells in MRS (after 30 or 120 min)/viable cells before lysozyme exposure × 100.

**Supplementary Table S3** Survival (log cfu/mL) of vaginal lactobacilli strains during *in vitro* gastrointestinal transit.

| Species | Strain | CI | SGJ | SIF |
| --- | --- | --- | --- | --- |
| *L. crispatus* (n=15) | C9 | 9.13±0.08 | 5.30±0.02 | 5.70±0.02 |
|  | D18 | 9.08±0.03 | 4.96±0.06 | 4.84±0.02 |
|  | D27 | 9.15±0.05 | 6.02±0.03 | 5.76±0.06 |
|  | E3 | 9.11±0.06 | 5.02±0.02 | 4.21±0.02 |
|  | G8 | 9.08±0.02 | 4.98±0.05 | 4.16±0.03 |
|  | J31 | 9.30±0.02 | 8.26±0.08 | 8.15±0.06 |
|  | J36 | 9.11±0.05 | 8.02±0.02 | 8.00±0.03 |
|  | N3 | 9.12±0.03 | 5.14±0.06 | 4.63±0.05 |
|  | S18 | 9.18±0.06 | 5.31±0.02 | 5.03±0.08 |
|  | T4 | 9.24±0.08 | 5.16±0.02 | 4.36±0.03 |
|  | U9 | 9.16±0.05 | 9.01±0.03 | 5.86±0.06 |
|  | V26 | 9.11±0.02 | 4.28±0.04 | 4.14±0.02 |
|  | Y11 | 9.13±0.03 | 4.05±0.02 | 3.83±0.02 |
|  | AB11 | 9.12±0.02 | 8.20±0.06 | 8.08±0.03 |
|  | AC7 | 9.21±0.03 | 8.11±0.02 | 8.03±0.02 |
| *L. gasseri* (n=15) | A9 | 9.12±0.03 | 8.25±0.02 | 8.03±0.02 |
|  | A14 | 9.06±0.06 | 8.15±0.04 | 7.95±0.03 |
|  | A18 | 9.13±0.08 | 8.21±0.03 | 8.12±0.02 |
|  | F5 | 9.18±0.02 | 8.24±0.02 | 8.21±0.04 |
|  | K2 | 9.18±0.04 | 5.02±0.02 | 4.56±0.06 |
|  | M9 | 9.10±0.03 | 4.34±0.02 | 4.46±0.02 |
|  | P5 | 9.09±0.04 | 4.21±0.08 | 4.13±0.04 |
|  | S21 | 9.03±0.06 | 5.07±0.06 | 5.15±0.02 |
|  | T13 | 9.02±0.03 | 5.10±0.02 | 4.60±0.04 |
|  | U5 | 9.00±0.02 | 4.80±0.02 | 4.28±0.08 |
|  | W14 | 9.24±0.04 | 8.21±0.02 | 8.10±0.02 |
|  | W18 | 9.09±0.08 | 7.98±0.02 | 7.90±0.04 |
|  | Y13 | 9.11±0.04 | 5.01±0.03 | 4.67±0.07 |
|  | Z9 | 9.08±0.06 | 5.10±0.07 | 4.94±0.07 |
|  | AC3 | 9.13±0.08 | 5.04±0.07 | 4.69±0.04 |
| *L. salivarius* (n=11) | C21 | 9.18±0.06 | 5.06±0.07 | 4.12±0.08 |
|  | D20 | 9.13±0.06 | 5.02±0.04 | 4.83±0.03 |
|  | G18 | 9.04±0.08 | 5.01±0.04 | 4.70±0.02 |
|  | H23 | 9.18±0.07 | 8.20±0.06 | 8.15±0.02 |
|  | M23 | 9.09±0.03 | 8.01±0.08 | 7.93±0.06 |
|  | N30 | 9.14±0.04 | 8.00±0.05 | 7.96±0.04 |
|  | O29 | 9.06±0.06 | 4.87±0.02 | 4.51±0.04 |
|  | P35 | 9.13±0.08 | 4.68±0.02 | 4.05±0.06 |
|  | T14 | 9.06±0.04 | 4.88±0.06 | 3.12±0.04 |
|  | Z15 | 9.17±0.03 | 9.03±0.06 | 8.91±0.03 |
|  | AD12 | 9.12±0.03 | 9.04±0.08 | 8.93±0.06 |
| *L. helveticus* (n=10) | C5 | 9.05±0.07 | 8.11±0.03 | 8.04±0.03 |
|  | G7 | 9.04±0.03 | 5.02±0.04 | 4.96±0.06 |
|  | G13 | 9.17±0.07 | 4.98±0.07 | 4.33±0.07 |
|  | P7 | 9.07±0.06 | 9.03±0.04 | 8.96±0.07 |
|  | P12 | 9.15±0.03 | 9.12±0.08 | 8.91±0.07 |
|  | S7 | 9.11±0.08 | 8.03±0.07 | 7.94±0.08 |
|  | T5 | 9.07±0.07 | 8.21±0.06 | 8.07±0.07 |
|  | U13 | 9.06±0.06 | 8.18±0.07 | 8.09±0.04 |
|  | Z3 | 9.14±0.03 | 8.54±0.08 | 8.31±0.03 |
|  | Z4 | 9.18±0.04 | 8.07±0.04 | 7.91±0.07 |
| *L. rhamnosus* (n=3) | E21 | 9.16±0.06 | 9.07±0.06 | 8.94±0.06 |
|  | L3 | 9.09±0.07 | 8.24±0.07 | 8.10±0.03 |
|  | L23 | 9.14±0.03 | 7.96±0.07 | 7.84±0.04 |
| *L. fermentum* (n=2) | E18 | 9.01±0.02 | 4.28±0.02 | 3.96±0.02 |
|  | AB2 | 9.05±0.02 | 4.23±0.06 | 4.11±0.08 |

Legend: Cell density before transit (CI) and upon sequential exposure to simulated gastric juice (SGJ) and simulated intestinal fluid (SIF) determined by viable plate count method on MRS medium. Data are shown as mean viable count on MRS agar from three independent experiments and standard deviation.

**Supplementary Table S4** Demographic characteristics of participating women.

| Demographic characteristics | |
| --- | --- |
| Sex (female, %) | 100 |
| Age (years ± SD) | 29.30 ± 6.11 |
| Weight (kg ± SD) | 63.15 ± 9.04 |
| Height (cm ± SD) | 170 ± 7.23 |
| BMI (kg/m2) | 21.8 ± 1.75 |

**Supplementary Table S5** Primer pairs used for species-specific PCR analysis.

| Set | Target | Primer pairs | Sequence (5’-3’) | Annealing T (°C) | Size of fragment | Reference |
| --- | --- | --- | --- | --- | --- | --- |
| 1 | *L. iners* | LinersF | GTCTGCCTTGAAGATCGG | 55 | 158 bp | [1S] |
|  |  | LinersR | ACAGTTGATAGGCATCATC |  |  |  |
| 2 | *L. crispatus* | LcrispatusF | TTACTTCGGTAATGACGTTA | 55 | 966 bp | [2S] |
|  |  | LcrispatusR | GGAACTTTGTATCTCTACAA |  |  |  |
| 3 | *L. gasseri* | LgasseriF | TCGAGCGAGCTTGCCTAGATGAA | 60 | 372 bp | [2S] |
|  |  | LgasseriR | CGCGGCGTTGCTCCATCAGA |  |  |  |
| 4 | *L. jensenii* | LjenseniiF | AGTCGAGCGAGCTTGCCTATAGAAG | 57 | 700 bp | [3S] |
|  |  | LjenseniiR | CTCACCCATCCGCCGCTAGCT |  |  |  |
| 5 | *L. acidophilus* | LacidoF | TGCAAAGTGGTAGCGTAAGC | 47 | 210 bp | [3S] |
|  |  | LacidoR | CCTTTCCCTCACGGTACTG |  |  |  |
| 6 | *L. plantarum* | LplantarumF | ATTCATAGTCTAGTTGGAGGT | 59 | 248 bp | [3S] |
|  |  | LplantarumR | CCTGAACTGAGAGAATTTGA |  |  |  |
| 7 | *L. fermentum* | LfermentumF | GCACCTGATTGATTTTGGTCG | 60 | 317 bp | [2S] |
|  |  | LfermentumR | GTCCATTGTGGAAGATTCCC |  |  |  |
| 8 | *L. salivarius* | LsalivariusF | CGAAACTTTCTTACACCGAATGC | 55 | 332 bp | [2S] |
|  |  | LsalivariusR | GTCCATTGTGGAAGATTCCC |  |  |  |
| 9 | *L. rhamnosus* | LrhamnosusF | TGCTTGCATCTTGATTTAATTTTG | 62 | 122 bp | [4S] |
|  |  | LrhamnosusR | GGTTCTTGGATYTATGCGGTATTAG |  |  |  |
| 10 | *L. reuteri* | LreuteriF | GGCGGCTGTCTGGTCTGCAA | 55 | 303 bp | [3S] |
|  |  | LreuteriR | GCTTGCGACTCGTTGTACCGTC |  |  |  |
| 11 | *L. paracasei* | LparacaseiF | CTAGCGGGTGCACTTTGTT | 60 | 312 bp | [2S] |
|  |  | LparacaseiR | GGCCAGCTATGTATTCACTGA |  |  |  |
| 12 | *L. helveticus* | LhelvF | CTGTTTTCAATGTTGCAAGTC | 60 | 500 bp | [5S] |
|  |  | LhelvR | TTTGCCAGCATTAACAAGTCT |  |  |  |

**SUPPLEMENTARY REFERENCES**

1. De Backer, E., Verhelst, R., Verstraelen, H., Alqumber, M. A., Burton, J. P., Tagg, J. R., et al. Quantitative determination by real-time PCR of four vaginal *Lactobacillus* species, *Gardnerella vaginalis* and *Atopobium vaginae* indicates an inverse relationship between *L. gasseri* and *L. iners. BMC Microbiol*. **7,** 115 [10.1186/1471-2180-7-115](https://doi.org/10.1186/1471-2180-7-115) (2007).
2. Byun, R., Nadkarni, M. A., Chhour, K. L., Martin, F. E., Jacques, N. A., Hunter N. Quantitative analysis of diverse Lactobacillus species present in advanced dental caries. *J. Clin. Microbiol*. **42**, 3128-3136 (2004).
3. Garg, K. B., Ganguli, I., Das, R., Talwar, G. P. Spectrum of *Lactobacillus* species present in healthy vagina of Indian women. *Indian J. Med. Res*. **129**, 652-657 (2009).
4. Ehrström, S., Daroczy, K., Rylander, E., Samuelsson, C., Johannesson, U., Anzén, B., Påhlson, C. Lactic acid bacteria colonization and clinical outcome after probiotic supplementation in conventionally treated bacterial vaginosis and vulvovaginal candidiasis. *Microbes Infect*. **12,** 691-699 (2010).
5. Petricevic, L., Domig, K. J., Nierscher, F. J., Krondorfer, I., Janitschek, C., Kneifel, W., Kiss, H. Characterisation of the oral, vaginal and rectal *Lactobacillus* flora in healthy pregnant and postmenopausal women. *Eur. J. Obstet. Gynecol. Reprod. Biol*. **160**, 93-99 (2012).
